# Urban Flora Biodiversity of Some Continental Cities of the Po Plain (Emilia-Romagna, Northern Italy)

**DOI:** 10.3390/plants14030450

**Published:** 2025-02-03

**Authors:** Alessandro Alessandrini, Michele Adorni, Fabrizio Buldrini, Sergio Montanari, Villiam Morelli, Mauro Pellizzari, Maurizio Sirotti, Giovanna Bosi

**Affiliations:** 1 Independent Researcher, 40018 San Pietro in Casale, Italy; 2 Orto Botanico—Sistema Museale di Ateneo, Università degli Studi di Parma, 43121 Parma, Italy; 3 Sistema dei Musei e Orto Botanico, Università degli Studi di Modena e Reggio Emilia, 41121 Modena, Italy; 4 Sistema Museale di Ateneo, Università di Bologna, 40126 Bologna, Italy; 5 Società per gli Studi Naturalistici della Romagna APS, 47121 Forlí, Italy; 6 Incia Soc. Coop., 42021 Bibbiano, Italy; 7 Liceo Scientifico “A. Roiti”, 44121 Ferrara, Italy; 8 Arpae—Sede di Forlí-Cesena, 47121 Forlí, Italy; 9 Laboratorio di Palinologia e Paleobotanica, Dipartimento di Scienze della Vita, Università degli Studi di Modena e Reggio Emilia, 41125 Modena, Italy

**Keywords:** ruderal species, therophytes, human disturbance, anthropogenic environments, floristic pollution, alien species, abandoned areas, urban periphery, global warming

## Abstract

Urban flora is a more and more interesting research subject, in light of the ongoing environmental change and biological homogenisation, since urban contexts are much more diversified that natural ones and, therefore, they offer much more colonisation possibilities to allochthonous species or unexpected refuges for endangered species. We have, therefore, added our own contribution by analysing the spontaneous vascular flora of 7 cities of the Emilia-Romagna Po Plain (northern Italy), one of the more culturally and economically developed areas in Europe. The global floristic list was 1305 species, spanning from 432 to 756 species in each individual city; 219 of them were constantly present in all cities examined. A notable richness in phytosociological classes (43 out of 75 known for the entire national territory) was observed. Therophytes were 35.4% of the spectrum, followed by hemicryptophytes, phanerophytes and geophytes. Eurasian and Mediterranean species dominated (average values 30 and 27%, respectively); exotic species were 26.8%, in line with the strong floristic pollution of Emilia-Romagna, with neophytes always prevailing over archaeophytes. Among neophytes, 44.8% came from the Americas and 26.2% from Asia. Some hydro-hygrophilous and halophilous species were found, owing to the presence of watercourses crossing the urban areas and coastal wetlands bordering one of the towns. The species new for the flora of Italy or Emilia-Romagna were 32, of which 24 were allochthonous. The species protected at a regional or national level were hardly present, which is normal in artificial environments. Despite the inevitable differences in exploration intensity and effort, this synthesis offers a picture of the contribution given by anthropogenic habitats to the global biological richness of the territory.

## 1. Introduction

Over the last few decades, spontaneous flora in cities has become a research subject raising more and more interest, which is understandable due to the numerous contributions on this topic. Urban development is rapidly transforming the world: it is not difficult to foresee that cities will continue to grow in population and expand their territory. Landscapes formerly characterised by agriculture and/or spontaneous vegetation will be altered and substituted by closed-up and impenetrable built-up areas [1]. In new environments such as these, vegetation is often restricted to small or very small patches which may continuously undergo drastic variations in ecological conditions [2,3]. Such changes are often advantageous for allochthonous flora, which is spreading heavily at the expense of native species, resulting in the extinction of many autochthonous species in urban areas [4] and the progressive development of new plant communities dominated by neophytes and generalist species.

Today, in most of the so-called developed countries, the flora of urban environments is generally characterised by a few native species and a great number of exotic ones, which arrived more or less recently through seeds and propagules. Among these adventitious species, we are able to distinguish those that arrived thanks to human dispersal, such as clothes, shoes, livestock, means of transport and food. Some of these were introduced deliberately (voluntary translocations), whereas others were introduced by accident [5]. The high level of propagule pressure typical of towns, indeed, greatly enhances the establishment of new entities (e.g., [6]); in addition, as is known, cities are rich in diversified habitats and growth environments for plant species because of the interaction of historical, socio-economic and cultural factors (e.g., [2,7,8,9]); therefore, they can host a much higher biological richness than the surrounding zones (e.g., [10]). For these reasons, cities often act as entry points through which alien species arrive into a new region and, potentially, naturalise in the territory (e.g., [11,12,13,14,15]), with connectivity, biogeography and climate being among the most important factors shaping the biological invasion process [16]. One of the effects of the translocations is the homogenisation of urban floras, which tend to have a notable contingent of species common to many cities [17,18]. Scientific literature concerning biotic homogenisation suggests that the selection of species with similar biological traits is promoted by common features of the towns: impervious surfaces, green area fragmentation and high disturbance levels due to human activities. Every city has its own local climate, historical development, types and density of infrastructures and amount of green areas, which interact generating different urban biotic communities in the diverse city sectors [19]; as a consequence, apart from some general characteristics, the specific features of single towns can differ notably from one city to another, which is reflected in the features of urban floras. The hypothesis of the uniformity of urban ecosystems, in fact, seems to better describe the typical situations of continental areas rather than those of more temperate zones (or, however, those with a more diversified flora), as shown by studies performed in Mediterranean Italy [20] and, more recently, Bosnia and Herzegovina [21]. In general, species composition seems to be much more influenced by habitat type than climate [22]. Given the great variety of study methods used across Europe to approach this kind of investigations, it is still too early to draw precise conclusions about many parts of the continent, especially southern ones [23].

Throughout the global movement of the species, we can recognise changes in three steps [24,25]: around 1500 (post-Mediaeval period), around 1850 (industrial revolution), around 2000 (globalisation). Each of these periods can be associated with a quantitative and qualitative leap in plant species translocation between continents [26].

There are many species introduced on purpose in various territories, particularly in cities, for example, species of agricultural, horticultural, ornamental or forestry interest (when they become part of the spontaneous flora, they are called ergasiophytes, *sensu* Di Castri et al. [27]), but the number of those which arrived accidentally is much higher.

Analyses of urban flora categorise plant species into native (i.e., belonging to the originary flora), archaeophytes (i.e., species introduced before wide geographical discoveries) and neophytes (species arrived recently); the results show a clear predominance of neophytes, whereas archaeophytes are more numerous in smaller cities [28]. In general, exotic species seem to spread out more in urban areas compared to the surrounding territories (e.g., [29,30]), but the presence of archaeophytes tends to decrease over time, especially as a consequence of more intense land use (e.g., [31,32]). Nonetheless, in 19 cities of the Urals and Volga region (European Russia), biotic homogenisation is given by archaeophytes [33], in contrast to what is usually found in western Europe. A further aspect worthy of investigation is the presence of apophytes, i.e., native species which can adapt to the ecological conditions of artificial environments, a phenomenon that is becoming more and more frequent in recent times [34].

Many contributions refer to the comparison of floras among diverse urban areas, trying to detect common trends and differences between the analysed zones [20,35,36]. Another interesting aspect is cities’ contribution to local biodiversity, which confutes the concept of the “biological desert” usually applied to urban areas in comparison to surrounding agricultural ones [37]. In any case, one of the most dangerous effects of urban sprawl and land use intensification (especially for agricultural uses) is the loss of species and habitats with respect to the native biological heritage, as confirmed by various authors (e.g., [9,38]). In the territory investigated, this fact is particularly evident when thinking of aquatic species, which have strongly decreased in the last century because of the negative impact of urbanisation on native hydro-hygrophytes (e.g., [39,40]).

A particular aspect of cities’ flora is the flora of the railway areas, which are of great importance for urban environments, even if they are difficult to explore [41,42].

In general, the predominance of therophytes is evident in urban ecosystems, having clearly adapted to high-stress conditions. Hemicryptophytes and phanerophytes follow, among which are numerous species introduced as ornamental (e.g., [43]). From a chorological viewpoint, in biogeographically similar situations, one can discover notable analogies among the floras of different towns of diverse countries. At least on a European scale, the allochthonous and (sub)cosmopolitan usually dominate, followed by European-Eurasian and euri- and steno-Mediterranean [44].

In addition, in some cases an analysis of the urban environmental conditions, particularly for soil nutrients, moisture and pH, was performed through Ellenberg’s bioindication indices (see, e.g., [36]; the case of Rome was analysed for the Italian territory [45]). The incidence of salinity, instead, is scarcely or not at all studied, even if in some cases it could be particularly expressive.

Despite the recent publication of an inventory of the Italian syntaxa dominated by allochthonous species (therefore also plausible for urban environments), which is important to put the results of floristic analyses in a phytosociological context [46], this kind of investigation has actually been performed quite rarely in urban ecosystems, both in Italy and other countries (see, e.g., [47]).

**Figure 1:**
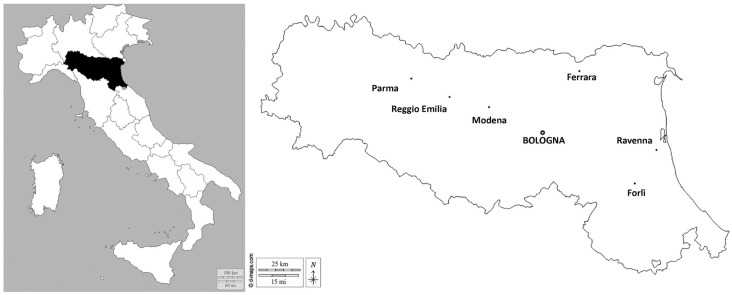
The cities studied and their position in Emilia-Romagna (map available online at https://d-maps.com/carte.php?num_car=22179&lang=it [accessed on 10 November 2024], with modifications).

This article gives a first synthesis of the floristic information available for 7 cities (Bologna, Ferrara, Forlí, Modena, Parma, Ravenna and Reggio Emilia; see Table 11 in the Materials and Methods) of one of the most culturally and economically developed European regions: the southern Po valley in Emilia-Romagna (northern Italy), which is one of the most densely populated areas on the continent, constantly frequented, colonised and exploited by humanity since prehistoric times (Figure 1; see Materials and Methods for further details). Until today, similar syntheses have been performed on some north-eastern Italian cities [35,48] or a few continental and Mediterranean cities [20]; on a European scale, there are comparable works in Germany [28], the Balkan Peninsula (e.g., [21,22,43]) and European Russia [33]. At a national level, Emilia-Romagna is the transition point between the Mediterranean subrealm, that covers the Italian peninsula and has a tendentially Mediterranean vegetation, and the Western Eurasia realm, that covers northern Italy and has a continental vegetation of central European or alpine type (see [49]); therefore, our analysis can represent an interesting case-study, given the coexistence of these two vegetation types.

The evolution of urban flora throughout the history of a city is a fascinating but still scarcely discussed topic. In Italy, partial studies have been carried out in some of the towns which are the subject of this article (Modena, Ferrara, Parma—see beyond) and in Florence [50]. More in-depth research has been published for Central European cities, sometimes using mainly archaeobotanical data (e.g., [51,52]) or information obtained from written sources and herbals (e.g., [8,9,53]).

To contribute to the debate in progress on urban ecology and the characteristics of cities as ecosystems, in this article we tried to:

- Present a reasoned synthesis of the data collected in recent years concerning the spontaneous flora of the 7 towns mentioned above, delineating some trends, common features or peculiar traits, given the climate similarity, but also the considerable differences in terms of history, population, industrial development and surrounding territory,
- Illustrate that, in the so-called developed countries, the great ecological and environmental diversity of cities hosts a larger floristic diversity than the surrounding extra-urban territory, spanning from generalist to rare protected species.

## 2. Results

### 2.1. General Characters

The total floristic list of the 7 towns consists of 1305 taxa, including species and subspecies (Table S1; hereafter, in the text we shall use the term “species” to refer to true species, subspecies or fixed hybrids). The highest diversity was recorded in Forlí and the lowest in Parma (Table 1). The correlation between number of species recorded and number of inhabitants is weak (ρ = 0.327); the correlation between number of species recorded and number of contributors (see Table 12) is moderate (ρ = 0.542).

The 1305 species are ascribed to 599 genera and 123 families, of which the most numerous are *Asteraceae* (*Compositae*; 11.0%), *Poaceae* (*Gramineae*; 10.2%), *Fabaceae* (*Leguminosae*; 7.0%), *Brassicaceae* (*Cruciferae*; 4.2%), *Lamiaceae* (*Labiatae*) and *Rosaceae* (both 4.1%), *Caryophyllaceae* (2.9%), *Apiaceae* (*Umbelliferae*) and *Amaranthaceae* (both 2.8%), *Cyperaceae* and *Plantaginaceae* (2.4%); 93 families account for less than 10 species each. The genera with most species are *Euphorbia* (20 species), *Trifolium* (19), *Carex* (16), *Allium* and *Veronica* (14 species each), *Amaranthus* (13), *Ranunculus* (12), *Juncus* and *Prunus* (11 species each), *Crepis*, *Rumex*, *Sedum* and *Vicia* (10 species each); all the remaining families have 9 species or less. Furthermore, 102 genera contain 2 species and 356 only 1.

#### 2.1.1. Rarity of Species and Affinity to Urban Environment

The rarity of the species can be summarised as follows:

- 428 species were found in 1 city
- 201 species were found in 2 cities
- 155 species were found in 3 cities
- 110 species were found in 4 cities
- 96 species were found in 5 cities
- 94 species were found in 6 cities
- 219 species were found in 7 cities

Considering the affinity to urban environments (“urbanophily”), from the total list of 1305 species, 428 can be regarded as steno-urbanophobous, 356 urbanophobous, 110 urban-neutral, 190 urbanophilous and 219 steno-urbanophilous.

#### 2.1.2. Biological Spectrum

**Figure 2:**
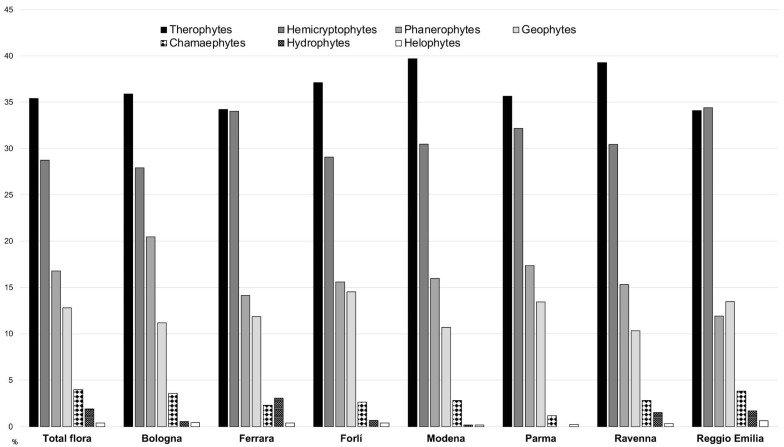
The life form spectra for the cities studied (the values are expressed as percentages); the spectrum calculated for the global flora (i.e., the entire floristic list of all cities, 1305 species) is also shown.

The life form spectra are reported in Figure 2. Annual species always dominate (34–39% of the spectrum), followed by hemicryptophytes and phanerophytes; in some cases, a discrete presence of hygrophilous species is also observed (never exceeding 5%, however). In two cases (Ferrara and Reggio Emilia), therophytes and hemicryptophytes are nearly equivalent in percentage.

No particular trend can be detected from the seaside towns towards the inland ones.

#### 2.1.3. Chorological Spectrum

**Figure 3:**
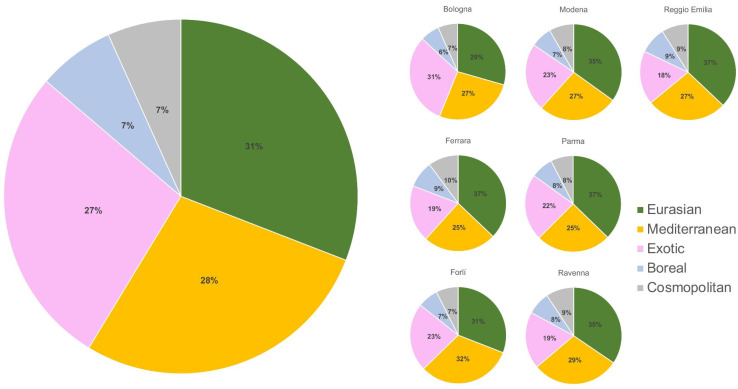
Chorological spectra for the cities studied; the spectrum calculated for the global flora (i.e., the entire floristic list of all cities, 1305 species) is also shown. Only the chorotypes with a number of species >3% are shown.

The chorological spectra are reported in Figure 3. Eurasian species nearly always dominate, followed by Mediterranean and exotic ones (only in Bologna the exotic are the most represented category); cosmopolitan and boreal species are practically equivalent.

At least apparently, one can distinguish cities with a warmer climate, given the higher presence of thermophilous species, and cities with a more continental climate, marked by the predominance of Eurasian species: in the first group there are Forlí and Ravenna, where Mediterranean species are 29–32% of the list; Parma and Ferrara are in the second group, where Eurasian species are 37% of the list and Mediterranean species are ca. 25%. Bologna, Modena and Reggio Emilia are somewhere in between.

#### 2.1.4. Phytosociological Spectrum

**Figure 4:**
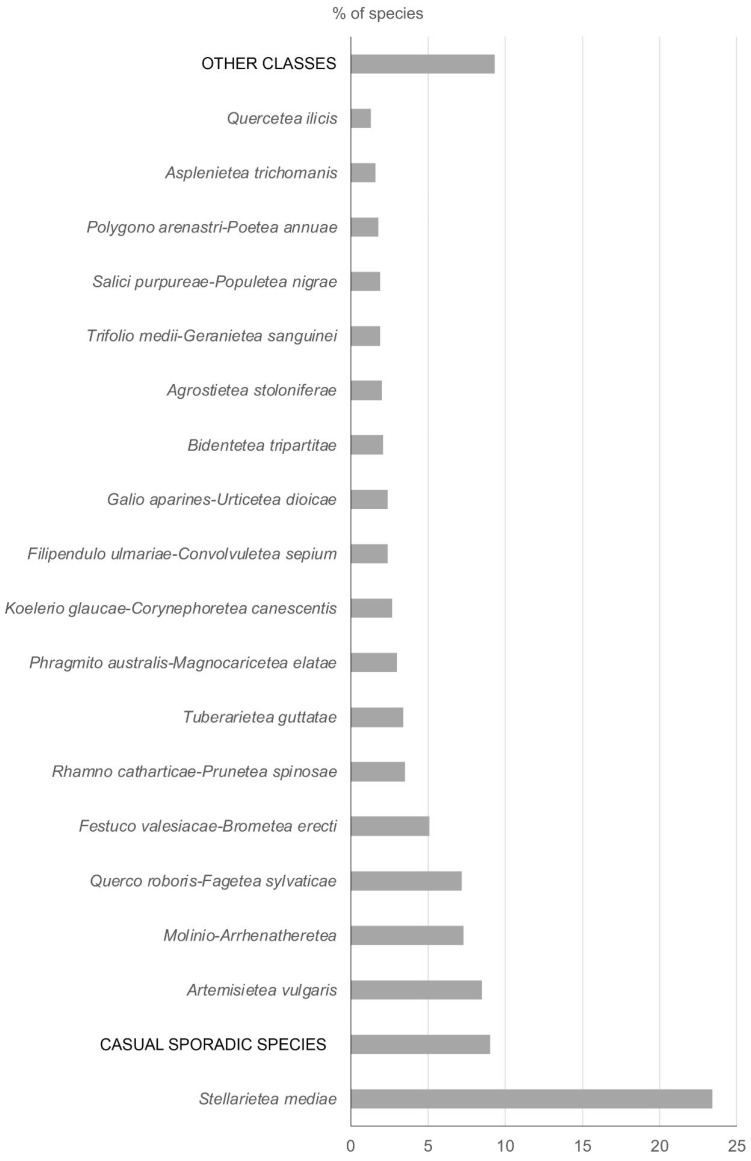
The phytosociological spectrum for the flora of the cities studied.

Urban species are subdivided into 43 classes, to which the “casual sporadic” should be added (Figure 4); 18 classes contain 90.7% of the total flora, among which are most classes of ruderal, synanthropic and/or nitrophilous vegetation (e.g., *Galio-Urticetea*, *Stellarietea mediae*, *Artemisietea vulgaris*, *Polygono-Poetea*). More generally, urban species may be sorted into 8 physiognomic macro-categories (see Table 2).

As for the frequency of the physiognomic macro-categories (Table 3), synanthropic species always dominate, followed by grassland and nemoral ones (the contribution of the remaining macro-categories is modest). Comparing the frequencies calculated for every city, Ravenna is the only one with a considerable presence of halophilous/psammophilous species, and Ferrara the only one with a considerable presence of aquatic species. Bologna has the highest percentage of chasmophytic/glareicolous and nemoral flora, Reggio Emilia the highest percentage of amphibian species, and Parma the highest percentage of synanthropic species.

**Figure 5:**
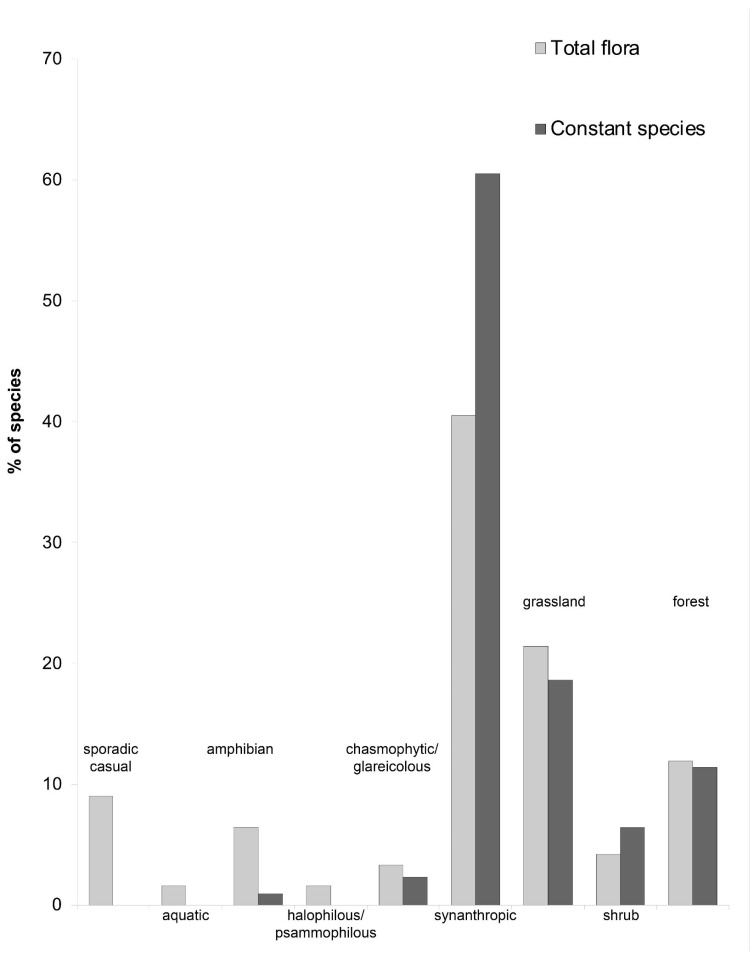
The incidence of the physiognomic macro-categories of vegetation for the cities studied, calculated for the global flora and the constant species (i.e., steno-urbanophilous ones).

Considering only constant species (i.e., the steno-urbanophilous), the incidence of synanthropic species increases considerably compared to the global flora (+20%, see Figure 5); the presence of grassland and nemoral species is also worth noting. Aquatic, halophilous/psammophilous and casual sporadic species are totally lacking, instead, and amphibian ones are drastically reduced.

Analysing the frequency of the 7 classes of synanthropic vegetation, both for the global flora and for the constant species only (Table 4), the most represented classes are *Stellarietea mediae* and *Artemisietea vulgaris*; the others follow at a notable distance. The differences between the global flora and constant species are not very pronounced. The same can be said when comparing the classes of synanthropic vegetation (Table 5) for every city analysed and the global flora.

Analysing the frequency of the 7 classes of grassland vegetation, both for the global flora and for the constant species only (Table 6), the most represented class is *Molinio-Arrhenatheretea*. Among the constant species, *Agrostietea stoloniferae* is more abundant than in the total flora, whereas *Festuco valesiacae-Brometea erecti* and especially *Tuberarietea guttatae* are less abundant. Comparing the classes of grassland vegetation for every city analysed and the global flora (Table 7), the most relevant differences concern *Festuco-Brometea* and *Tuberarietea guttatae*: the latter class, composed of Mediterranean thermophilous species, seems to show a decreasing trend moving from eastern cities (except for Ferrara) to western ones.

#### 2.1.5. Species Protected on a Regional, National or International Level

**Scheme 1:**
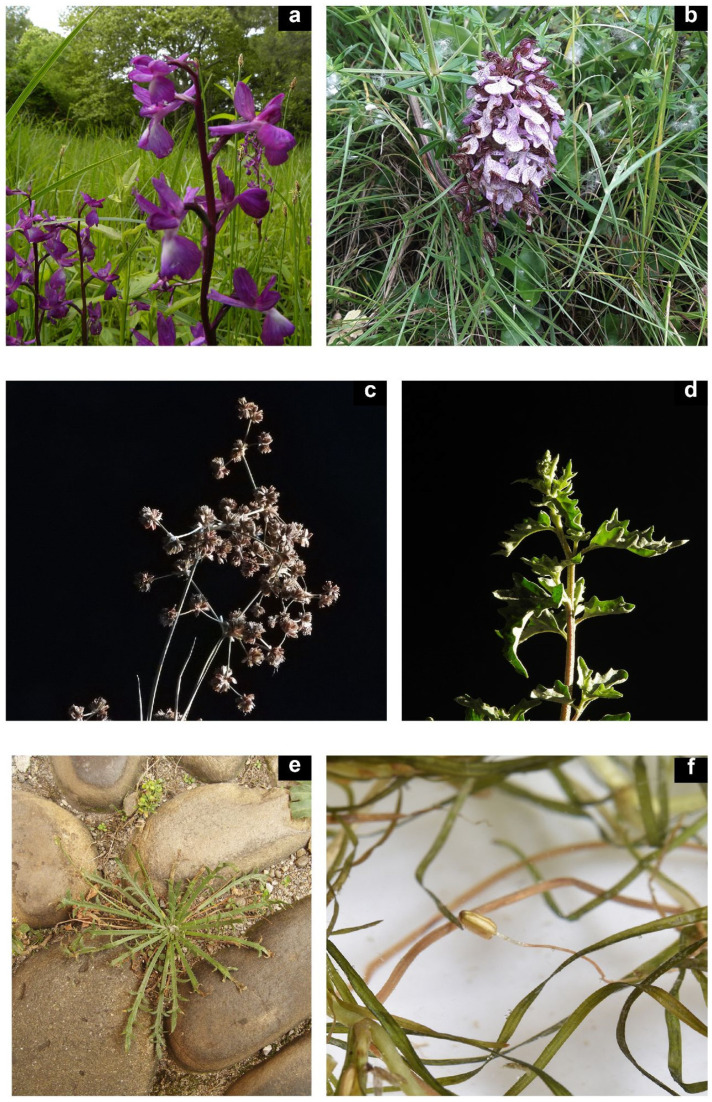
Some examples of protected species: (**a**) *Anacamptis laxiflora* (Lam.) R.M. Bateman, Pridgeon et M.W. Chase (Bologna); (**b**) *Orchis purpurea* Huds. (Ferrara); (**c**) *Juncus subnodulosus* Schrank (Modena). Two examples of halophilous species: (**d**) *Atriplex tatarica* L. (Modena), (**e**) *Plantago coronopus* L. (Modena). Hydro-hygrophilous species: (**f**) *Zannichellia peltata* Bertol. (Bologna). Photographs: A. Alessandrini (**a**,**c**,**d**,**f**), F. Buldrini (**e**), M. Pellizzari (**b**).

The present study revealed the presence of 33 species of interest from a conservation perspective and protected on a regional or national level (Scheme 1a–c), such as *Galanthus nivalis* L., *Juncus subnodulosus* Schrank, *Narcissus tazetta* L., *Nymphaea alba* L. or various orchids (*Anacamptis* spp., *Cephalanthera damasonium* (Mill.) Druce, *Listera ovata* (L.) R. Br., *Neotinea tridentata* (Scop.) R.M. Bateman, Pridgeon et M.W. Chase, *Ophrys* spp., *Orchis* spp.).

### 2.2. Steno-Urbanophilous Species (i.e., Species Present in All of the Cities Considered Here)

**Scheme 2:**
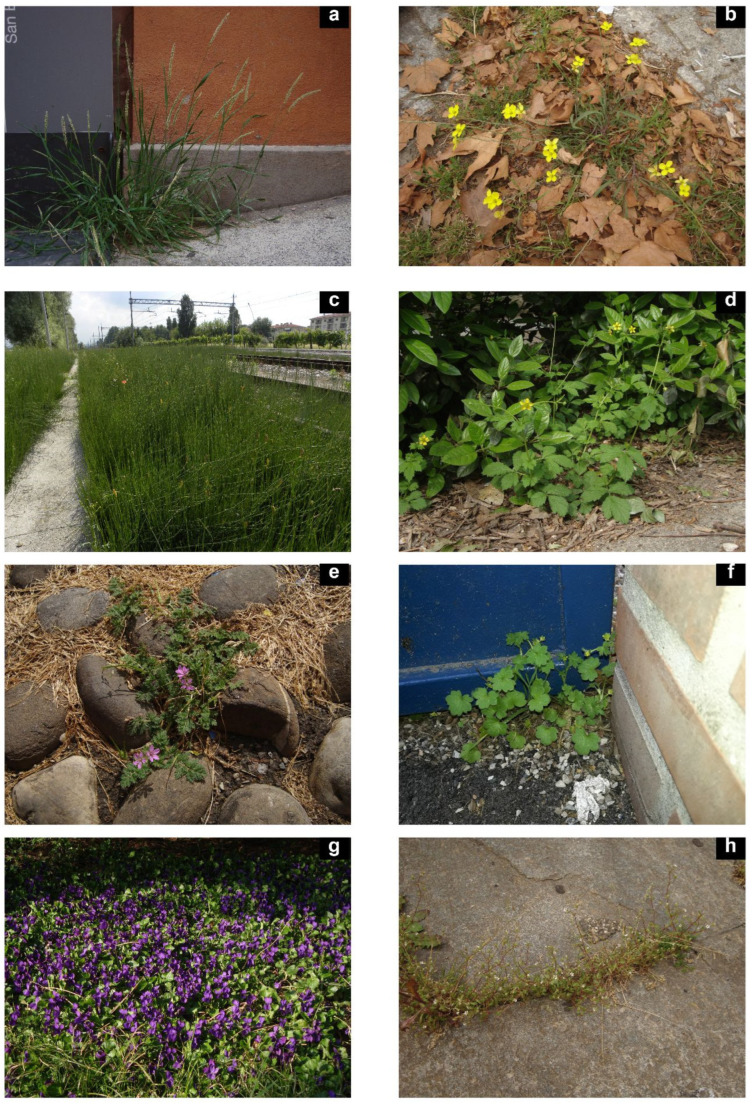
Some steno-urbanophilous species seen in their growth environments: (**a**) *Alopecurus myosuroides* Huds. (Modena), (**b**) *Diplotaxis tenuifolia* (L.) DC. (Modena), (**c**) *Equisetum ramosissimum* Desf. (Bologna), (**d**) *Geum urbanum* L. (Modena), (**e**) *Erodium cicutarium* (L.) L’Hér. (Modena), (**f**) *Ranunculus parviflorus* L. (Ferrara), (**g**) *Viola odorata* L. (Modena), (**h**) *Saxifraga tridactylites* L. (Modena). Photographs: A. Alessandrini (**c**), F. Buldrini (**a**,**b**,**d**,**e**,**g**,**h**), M. Pellizzari (**f**).

**Scheme 3:**
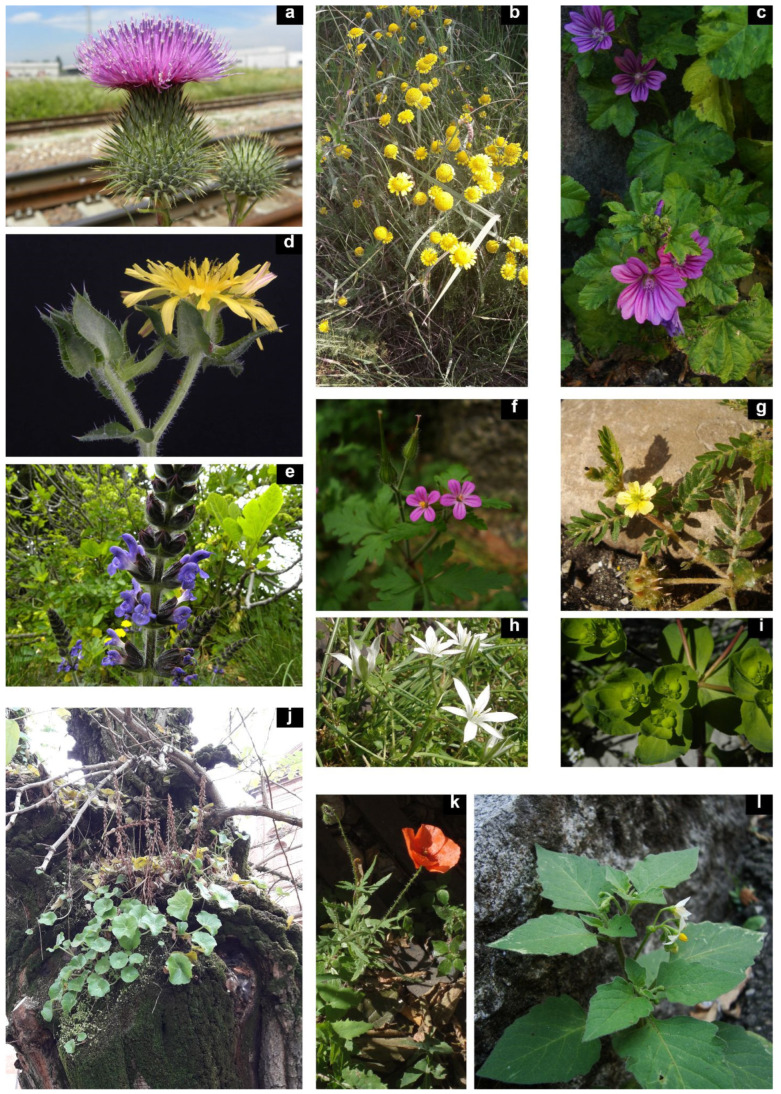
Some examples of steno-urbanophilous species: (**a**) *Cirsium vulgare* (Savi) Ten. (Bologna), (**b**) *Cota tinctoria* (L.) J. Gay (Ferrara), (**c**) *Malva sylvestris* L. (Modena), (**d**) *Helminthotheca echioides* (L.) Holub (Bologna), (**e**) *Salvia verbenaca* L. (Bologna), (**f**) *Geranium purpureum* Vill. (Modena), (**g**) *Tribulus terrestris* L. (Modena), (**h**) *Ornithogalum divergens* Boreau (Modena), (**i**) *Euphorbia helioscopia* L. (Bologna), (**j**) *Umbilicus rupestris* (Salisb.) Dandy (Ferrara), (**k**) *Papaver rhoeas* L. (Modena), (**l**) *Solanum nigrum* L. (Modena). Photographs: A. Alessandrini (**a**,**d**,**e**,**i**), F. Buldrini (**c**,**f**,**g**,**h**,**k**,**l**), M. Pellizzari (**b**,**j**).

Globally, there are 219 steno-urbanophilous species (i.e., species present in all of the cities studied), i.e., 16.8% of the total floristic list (Scheme 2 and Scheme 3). About 40% are annual species, to which the 5–6% of biennial hemicryptophytes should be added: species with a short or very short life cycle are therefore 45% of the list. Phanerophytes, in many cases, are species typical of middle latitudes in the Old World (*Acer campestre* L., *Hedera helix* L., *Prunus spinosa* L., *Quercus robur* L., *Ulmus minor* Mill., etc.), not rarely cultivated as ornamental or food species and/or behaving as invasive (*Acer negundo* L., *Amorpha fruticosa* L., *Ailanthus altissima* (Mill.) Swingle, *Broussonetia papyrifera* (L.) Vent., *Gleditsia triacanthos* L., *Robinia pseudoacacia* L., etc.): out of a total of 48 species, 18 are cultivated and/or invasive.

Considering the chorotypes, Eurasian species make about 40% of the list, followed by Mediterranean ones (~25%); the Boreal species never surpass 9%.

In general, from an ecological viewpoint, all of the steno-urbanophilous species can be ascribed to one or more of these categories:

- Ruderal or synanthropic species;
- Grassland species, typical of polyphitic prairies with a certain disturbance or ruderality level;
- Woody species typically found in middle-latitude climates, used for street furniture (parks, tree-lined avenues, urban landscaping, etc.);
- Allochthonous species (ornamental, food plants, etc.) escaped from cultivation and naturalised in the territory.

### 2.3. Hydro-Hygrophilous Species

Globally, there are 213 hydro-hygrophilous species, i.e., 16.3% of the total floristic list (Scheme 1c–f). Among the hygrophilous species are, for example, *Equisetum* spp., *Alopecurus rendlei* Eig, *Carex tomentosa* L., *Centaurium pulchellum* (Sw.) Druce, *Frangula alnus* Mill.; among the palustrine species are, e.g., *Alisma plantago-aquatica* L., *Carex pseudocyperus* L., *Glyceria maxima* (Hartm.) Holmb., *Juncus fontanesii* J. Gay, *Oenanthe fistulosa* L.; among the hydrophilous species are *Ceratophyllum* spp., *Lemna* spp., *Hydrocharis morsus-ranae* L., *Nasturtium officinale* R. Br., *Potamogeton* spp., *Ranunculus trichophyllus* Chaix, etc.

Hydrophilous and palustrine species are generally more numerous in the cities crossed by watercourses (Table 8)—Ferrara, Forlí and Reggio Emilia—or with wetlands located within the urban area (as in the case of Ravenna).

### 2.4. Halophilous Species

Globally, there are 50 halophilous species, i.e., 3.8% of the total floristic list (Scheme 1d,e). Among the myo-halophytes are species like *Atriplex tatarica* L., *Bolboschoenus laticarpus* Marhold, *Lagurus ovatus* L., *Phragmites australis* (Cav.) Trin ex Steud., *Tamarix gallica* L.; the facultative halophytes include, for example, *Elymus acutus* (DC.) M.-A. Thiébaud, *Hainardia cylindrica* (Willd.) Greuter or *Sporobolus schoenoides* (L.) P.M. Peterson, growing on brackish soils; the true halophytes are *Halimione portulacoides* (L.) Aellen, *Limbarda crithmoides* (L.) Dumort., *Limonium narbonense* Mill., *Puccinellia festuciformis* (Host) Parl., etc., typical of coastal areas or salt lagoons.

Halophilous species show a clear tendency to decrease going inland from the coast (Table 9). The correlation between the number of halophilous species and the distance from the sea is worth noting: ρ = −0.668 for the facultative halophytes and ρ = −0.746 for the myo-halophytes. True halophytes were observed only in Ravenna, where they form 2.5% of the flora.

### 2.5. Allochthonous Species

**Scheme 4:**
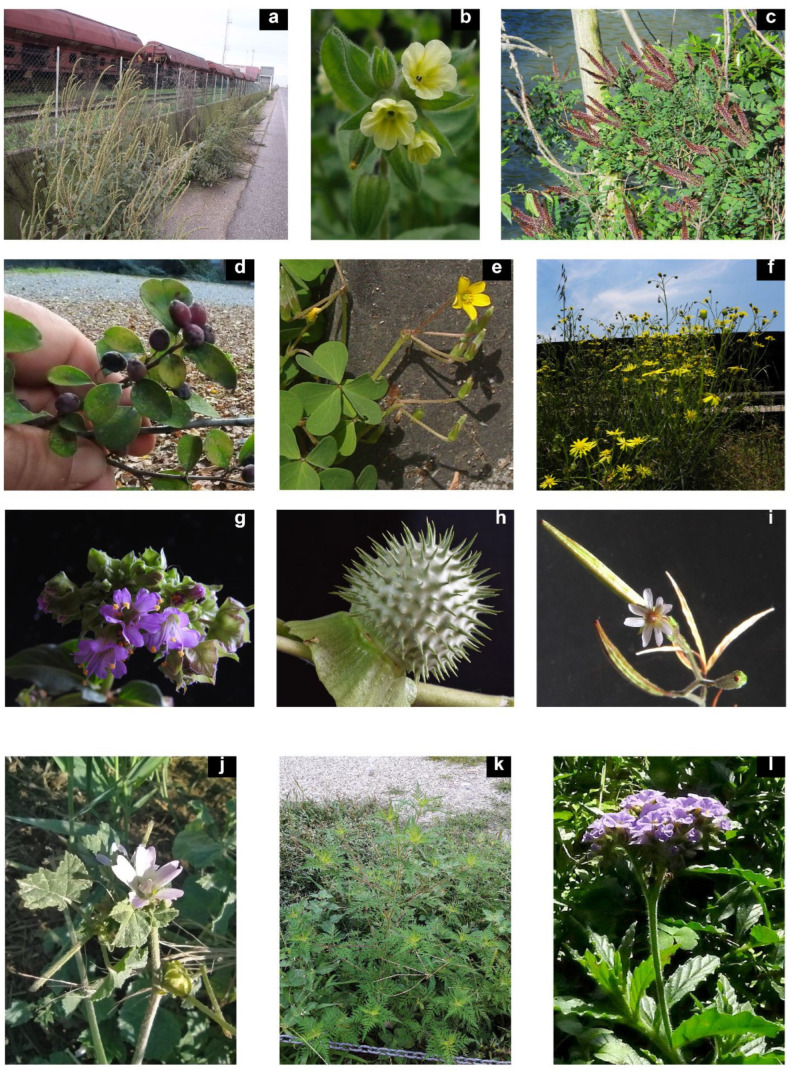
Some examples of allochthonous species: (**a**) *Amaranthus palmeri* S. Watson (Ravenna); (**b**) *Nonea lutea* (Desr.) DC. (Modena); (**c**) *Amorpha fruticosa* L. (Ferrara); (**d**) *Cotoneaster hissaricus* Pojark. (Bologna); (**e**) *Oxalis corniculata* L. (Modena); (**f**) *Senecio inaequidens* DC. (Bologna); (**g**) *Oxybaphus nyctagineus* (Michx.) Sweet (Bologna); (**h**) *Datura wrightii* Regel (Bologna); (**i**) *Epilobium brachycarpum* C. Presl (Bologna); (**j**) *Malva multiflora* (Cav.) Soldano, Banfi et Galasso (Ravenna); (**k**) *Ambrosia artemisiifolia* L. (Ferrara); (**l**) *Heliotropium amplexicaule* Vahl (Bologna). Photographs: A. Alessandrini (**d**,**f**,**g**,**h**,**i**,**l**), F. Buldrini (**b**,**e**), S. Montanari (**a**,**j**), M. Pellizzari (**c**,**k**).

Globally, there are 350 allochthonous species (archaeophytes + neophytes), i.e., 26.8% of the total floristic list (Scheme 4; Table S2); their influence on the flora of each town varies from 18.2% in Reggio Emilia to 30.9% in Bologna (Table 10). Archaeophytes always are a marginal presence with respect to neophytes.

Among the archaeophytes, we can find species cultivated for food from ancient times to the present day (e.g., *Atriplex hortensis* L., *Beta vulgaris* L., *Hordeum vulgare* L., *Prunus dulcis* (Mill.) D.A. Webb, *P. persica* (L.) Batsch, *Pyrus communis* L.), fodder species (e.g., *Medicago sativa* L., *Trigonella foenum-graecum* L.), oleiferous species (*Brassica napus* L.), ancient medicinal plants (e.g., *Calendula officinalis* L., *Galega officinalis* L., *Ricinus communis* L.), dyeing plants (*Isatis tinctoria* L.), textile fibre plants (*Cannabis sativa* L., *Linum usitatissimum* L.), commensal farmland species (e.g., *Abutilon theophrasti* Medik., *Centaurea cyanus* L.), spices (e.g., *Anethum graveolens* L., *Coriandrum sativum* L., *Origanum majorana* L.) and ornamental species (e.g., *Alcea rosea* L., *Cupressus sempervirens* L., *Hyacinthus orientalis* L., *Iris germanica* L.).

Among the neophytes, we can find species cultivated for food (e.g., *Capsicum annuum* L., *Cucurbita maxima* Duchesne, *Glycine max* (L.) Merr., *Zea mays* L.), fodder species (*Phacelia tanacetifolia* Benth.), oleiferous species (*Helianthus annuus* L.), species accidentally introduced by gardening (e.g., *Acalypha* spp., *Amaranthus* spp., *Eragrostis* spp., *Euphorbia* spp.) and many ornamental species (e.g., *Acer* spp., *Cedrus* spp., *Chimonanthus praecox* (L.) Link, *Cotoneaster* spp., *Elaeagnus* spp., *Ginkgo biloba* L., *Ipomoea purpurea* Roth, *Ligustrum* spp., *Solanum pseudocapsicum* L.).

**Figure 6:**
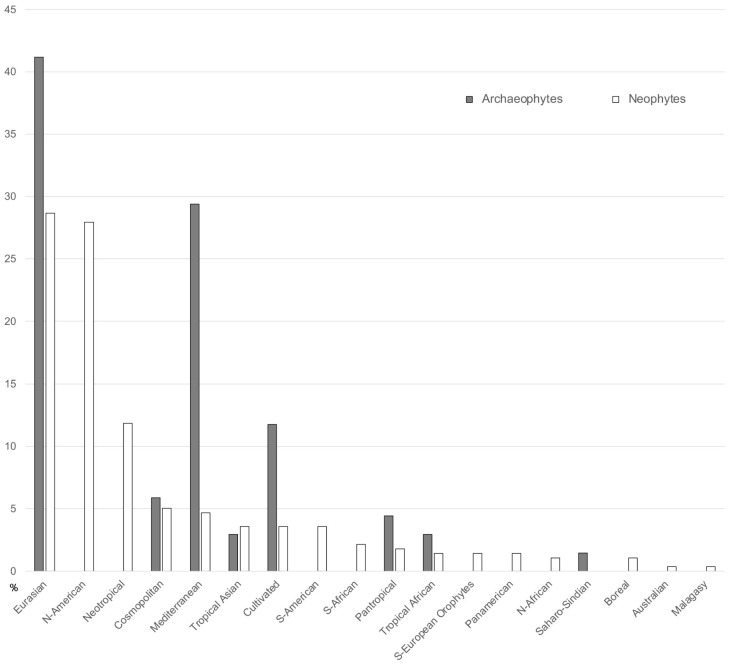
Chorological spectrum for allochthonous species.

The archaeophytes mostly come from Eurasia (Asia Minor, the Near East and central Asia, principally), and the Mediterranean basin, but also from the tropical regions of the Old World (11.8%; Figure 6).

Neophytes mostly come from the Americas (44.8%), but also from Asia (26.2%: species mainly native to the temperate and tropical zones of central and eastern Asia and the Indian subcontinent), with very limited apports from the Mediterranean basin (4.7%) and temperate Africa (3.2%). Tropical species (palaeotropical + neotropical) are 18.6% of the total flora. The increase in species coming from the warmest regions of the globe is 63.4% compared to what is observable among the archaeophytes.

### 2.6. Species New for the National or Regional Flora

The researches performed allowed us to discover 32 new species for the national or regional flora (Table S3).

In many cases, they were ornamental species (e.g., *Gazania* gr. *hybrida*, *Lobelia erinus* L., *Mazus pumilus* (Burm. fil.) Steenis [54,55], *Rubus laciniatus* Willd., *Tradescantia cerinthoides* Kunth, *Yucca recurvifolia* Salisb.), but we also found food species escaped from cultivations (e.g., *Capsicum annuum* L., *Ocimum basilicum* L., *Solanum melongena* L. [54]), exotic species introduced accidentally (e.g., *Amaranthus crispus* (Lesp. et Thévenau) A. Terracc., *Digitaria violascens* Link) and sometimes species native to Italy, but typically steno-Mediterranean (e.g., *Malva multiflora* (Cav.) Soldano, Banfi et Galasso, *Nerium oleander* L.). Railway yards revealed the presence of exotic species, such as *Bidens subalternans* DC., *Epilobium brachycarpum* C. Presl [56] or *Oxybaphus nyctagineus* (Michx.) Sweet [57,58], and native steno-Mediterranean species (*Ceratonia siliqua* L. [59]). In a few cases, in the urban sections of canals some new species were also found, like *Zannichellia peltata* Bertol. [60].

Globally, exotic species are 75% of the list of the new species.

## 3. Discussion

### 3.1. General Characters

This study analyses the urban flora of 7 cities of ancient foundation in the south-eastern part of the Po Plain, one of the most industrialised and economically developed European areas. Despite our results being far from definitive, we can, however, try to delineate some tendencies in order to contribute to the debate in progress on urban flora and its characteristics.

The cities analysed are different in terms of age of foundation, number of inhabitants, compactness of urban tissue, exploration intensity and environmental variety (see Table 11 in the Materials and Methods).

The total floristic list sums up to 1305 species, a considerable number, which is 37% of the floristic list of the entire Emilia-Romagna region, accounting for 3530 species and subspecies [61,62]; this richness is also visible in the high number of phytosociological classes detectable (43 out of the 75 defined for the entire Italian flora [63]) and the presence of almost all of the physiognomic macro-categories recorded in Italian vegetation (*sensu* [63]): the only macro-category not represented here is supra-sylvatic vegetation (Table 2). Among the cities, there are notable differences in the number of species recorded (Table 1), which are mostly due to the different exploration efforts, as can be deduced from the number of works published on this subject and the number of contributors (Table 12), at times extremely high if compared to the not-wide dimensions of the city itself. The case of Forlí is emblematic: it is the smallest town, but the number of species observed is the highest, probably because of the large amount of local people devoted to floristic research (also thanks to the illustrious tradition started by Pietro Zàngheri about a century ago: see, e.g., [64,65,66,67,68,69,70,71]) who meticulously analysed all of the environments distinguishable, even those not much considered generally, such as the car parks of supermarkets and commercial areas, where plants can grow in the gaps between interlocking tiles. In these places, particularly explored in Forlí and much more overlooked in the other cities, plants often assume dwarf forms, but they are still identifiable: here, in fact, species like *Stachys arvensis* (L.) L., *Campanula erinus* L. and *Lotus ornithopodiodes* L. were found, which are lacking in all of the other cities here considered, but whose presence is, however, plausible given the existence of these environments in all of the towns. The opposite case is that of Parma, the second largest city in Emilia-Romagna (Table 11): the number of species recorded is the lowest (Table 1), not because of a scarcity of growth environments, but rather because of the still insufficient exploration. Modena, which is similar to Parma in terms of number of inhabitants, revealed a flora of 606 species, despite the fact that—unlike Parma—it is not crossed by a river and not all of the built-up area was explored. Furthermore, some artificial environments, such as private courtyards and gardens and building roofs (especially tiled ones, typical of the historical centres), were not analysed; therefore, caution is needed in evaluating the real consistency of the urban flora. Given these premises, it is not unreasonable to think that the true number of species which are present in every city is actually higher than that recorded, as already supposed for the town of Modena [54].

Many of the most represented families on a national scale are among the most represented in the major towns with nearly equivalent percentages [72,73,74], as is also seen for those most represented on a European level (e.g., [41,43]). In the cities analysed, annual or however herbaceous species always dominate (Figure 2), on average composing 75% of the flora (of which 35–40% are therophytes). This is in line with the climatic and pedological features of artificial environments, which are tendentially hot, arid and sterile and subjected to a more or less intense trampling (e.g., [75,76,77]): not by chance, species like *Poa annua* L., *Polygonum rurivagum* Jord. ex Boreau, *Portulaca oleracea* L., *Polycarpon tetraphyllum* L., *Stellaria media* L. and *Cichorium intybus* L. are frequent in all of the cities analysed and are also listed among the most common species in Italian urban ecosystems [78]. From a phytosociological perspective, indeed, the most frequent classes are those linked to ruderal and disturbed environments [79], such as *Stellarietea mediae* Tüxen, Lohmeyer & Preising ex Von Rochow 1951 and *Artemisietea vulgaris* Lohmeyer, Preising & Tüxen ex Von Rochow 1951 (Figure 4, Table 4), which are substantially constant in the cities analysed (Table 5). Not by chance, the physiognomical category of the synanthropic species is much more represented than all of the others, accounting for almost half of the entire floristic list (Table 3). Considering the grassland vegetation, the dominant classes are *Molinio-Arrhenatheretea* Tüxen 1937 and *Festuco valesiacae-Brometea erecti* Br.-Bl. & Tüxen ex Br.-Bl. 1949 (Table 6 and Table 7), i.e., those including some of the most common grassland types in the middle latitudes of the Old World in continental areas. In particular, the latter class represents the zonal steppe vegetation of the Russian plain, whereas towards the western part of Eurasia it includes secondary grasslands with tendentially dry soils or with rapid drainage [79]; to the *Molinio-Arrhenatheretea* are ascribed communities of artificial pastures, meadows and meadow fringes, with fertile soils, generally at low altitudes [79]. Translating these features to the urban environment, we can easily detect the communities of arid soils such as sett or cobblestone paving, the backfill of uncultivated flowerbeds, etc., and those of lawns and landfill or loose earth in the largest flowerbeds along the boulevards. Phanerophytes are, however, relatively abundant (at least in terms of number of species): in various cases they are nemoral or pre-forest species (Table 2) native to Italy, whereas in many others they are ornamental species escaped from cultivation which could reproduce and spontaneously spread. The progressive adaptation of the allochthonous species to the urban climate and environment, with the selection of genotypes able to resist and profitably grow and proliferate in these conditions, was already demonstrated, indeed, in [80]. This tendency is also shown by the notable presence of exotic species (Figure 3), which is around 25% of the total flora, but in Bologna surpasses 30%, very probably because of the much larger dimensions of the city compared to all of the others and its great importance as a railway and route hub for the entirety of northern Italy. Among autochthonous species, the Eurasian, Mediterranean and cosmopolitan together make up more than 60% of the chorological spectrum, whereas boreal species reach 10% at most, thus confirming the thermophilous tendency of urban flora. This fact is also marked by the geographical distribution of the exotic species (Figure 6): the palaeotropical, neotropical and pantropical together form 21.5% of the exotic flora (archaeophytes plus neophytes) and 4.6% of the total flora of the 7 cities, a notable result considering that we are speaking of an area located around 45° latitude and with a continental climate (Table 11; [81,82]). More generally, macrothermal species (Mediterranean, tropical and thermo-cosmopolitan) form 31.8% of the total flora, similarly to what was observed in the historical city centre of Modena [54], with a very pronounced difference compared to the reference province (about 20% of species being macrothermal [83]), but only a slight one when thinking of the sole plain area of this province (ca. 29% of species being macrothermal [83]), which can be assumed to be representative of the average conditions of Emilia-Romagna for its central position. As already seen, in fact, the differences are expressed more in the quality than the quantity of the chorotypes, with a predominance of euri-Mediterranean species in the plain and steno-Mediterranean and tropical ones in the cities, because of the steppe characteristic of the urban environment [84]. It is, however, clear that the urban heat island effect is one of the main causes of thermophilous species being significantly more present in the cities’ flora than in the surrounding territory.

Concerning the species of conservation interest, all of them were recorded in urban grasslands or wetlands, woodland strips or urban parks, as already seen in other studies, thus confirming the notable species and environmental richness typical of cities [85,86], even small ones. Nonetheless, their presence is more than marginal if compared to the total flora (2.5% of the entire list), which is not surprising given that cities are places subjected to a continuous and heavy disturbance and protected species generally are the most exigent from an ecological viewpoint [54]. Many of them actually behave as somehow ruderal or pioneer species, such as *Anacamptis pyramidalis* (L.) Rich., *Orchis purpurea* L., etc.; others are typically nemoral or scyaphilous (*Galanthus nivalis* L., *Cephalanthera damasonium* (Mill.) Druce, *Listera ovata* (L.) R. Br.), whereas some entities (*Ilex aquifolium* L., *Taxus baccata* L.) are ornamental species escaped from cultivation which have spontaneously spread out into the surrounding territory [87]. Hydro-hygrophilous species were also present, even if sporadically, recalling the ancient richness of humid environments on the Po Plain (see, e.g., [88,89,90,91,92]). Other species, despite not being protected, are, however, rare and worthy of attention: in most cases they also are hydro-hygrophilous, such as *Euphorbia palustris* L., *Gratiola officinalis* L., *Nymphoides peltata* (S.G. Gmel.) O. Kuntze and *Typha angustifolia* L., which are currently confined in circumscribed places, not only for their ecological features, but also due to the more and more intense human impact on the regional territory [40,93].

### 3.2. Steno-Urbanophilous Species (Species Present in All of the Cities Considered Here)

Steno-urbanophilous species comprise 16.8% of the total species list. They are typical of very disturbed and dynamic environments, as demonstrated by the high presence of therophytes and biennial species (*circa* 45% of the steno-urbanophilous list) and, more generally, by the auto-ecology of these species, which always have a certain degree of ruderality [77]. Among life forms, therophytes are dominant because they are adapted to dry, hot, unstable environments not rarely trampled, which often characterise the urban ecosystem [20,94]. This fact is in line with what was observed in 11 cities in the Republic of Serbia [43], even if with different percentages of species common to all of the towns (7.1% of the total list *versus* 16.8%). Similar results were also found in cities with a not necessarily continental climate: 10.8% among 4 cities of central and southern Italy [20], 8.8% among 7 cities of Bosnia and Herzegovina [21]. Therefore, this 16.8% of steno-urbanophilous species recalled above reveals a certain similarity (at least in the fundamental traits) with the urban floras of central European settlements, where the steno-urbanophilous are normally 15% of the total floristic list [95].

It is interesting to note that 84% of the steno-urbanophilous species are common to both the 7 cities of Emilia-Romagna and the 11 Serbian cities mentioned above, which means that the urban ecosystem shows a certain constancy in some general characters so that the most frequent species are mostly the same, even in non-bordering countries. The exotic species percentages also are perfectly comparable: 13% of the steno-urbanophilous species in the 11 Serbian cities [43] and 15.9% in the 7 cities of Emilia-Romagna, thus marking the notable floristic pollution of this Italian region [62].

From a phytosociological viewpoint, there are no particular differences between the entire flora and the constant species (the steno-urbanophilous species) when considering the synanthropic vegetation classes (Table 4); on the contrary, some variations are visible in the grassland vegetation (Table 6), with *Agrostietea stoloniferae* much more represented than in the total flora (19.5% *versus* 9.3), like *Molinio-Arrhenatheretea*, whereas *Tuberarietea guttatae* strongly decreases (4.9% *versus* 15.9). When reasoning about the distribution of the classes in the 7 cities, the latter clearly marks the progressive transition from more Mediterranean and mild conditions in the eastern cities (save Ferrara) to a more continental and rigid climate in western ones. This class is in fact formed by Mediterranean thermophilous ephemeral species typical of annual communities on acidic soils [79], i.e., conditions more easily found in Forlí and Ravenna, due to the proximity of the Adriatic Sea, than in Parma or Reggio Emilia.

Nonetheless, a methodological precisation is needed: we assigned the species to a given “urbanophily” category based on a simple statistical criterion, that is, the number of cities where they were observed. In fact, currently there is not a precise way to evaluate species affinity to the urban environment, because this would require auto-ecological analyses which are lacking for many of the entities that may be found in urban ecosystems. For several of them, a first evaluation is already available (e.g., [35,96,97]), even if it is based on an expert’s assessment; in the remaining cases, the only affordable method is the authors’ own experience. For these reasons, and for obvious uniformity exigencies, we preferred the statistical approach recalled above. Despite this, our interpretation does not differ particularly from that provided by Buccheri and Martini [35], at least in the final evaluation of the single species, because steno-urbanophilous (as circumscribed in our work) species are often annual and thermophilous, although phanerophytes may also be present, such as *Broussonetia papyrifera* (L.) Vent., *Ficus carica* L., *Laurus nobilis* L., etc. Finally, it must be remembered that a certain number of urbanophilous species, which were currently found in 6 cities out of 7, could also be observed in all of the towns investigated if *ad hoc* research were performed: the urbanophilous species are ruderal and generalist, too, even if they are not strictly linked to urban environments only. Therefore, the steno-urbanophilous category might reach, and even surpass, 20% of the total floristic list.

### 3.3. Hydro-Hygrophilous Species

In urban contexts, the presence of hydro-hygrophilous species is generally uncommon and linked to the eventual presence of a watercourse within the urban tissue, given also that surface waters have notably decreased in recent decades because the canals (a typical element of many cities of the Po Plain) in the urban section have been covered over [98]. Furthermore, these species may not be observed at all if a particular study method is adopted, that is, systematically excluding all of the not strictly artificial environments [99]. Nonetheless, the common practice of watering flowerbeds and lawns allows some hygrophilous species to grow, as in the cases of *Althaea officinalis* L., *Eupatorium cannabinum* L., *Mentha* spp., *Scirpoides holoschoenus* (L.) Soják, etc., whose presence is related to the irrigation (at times even hyper-irrigation) of flowerbeds, traffic islands, roundabouts, etc. [98]. Other species, instead, which are true hydrophytes or helophytes (e.g., *Alisma* spp., *Bidens* spp., *Ceratophyllum* spp., *Lemna* spp., *Typha* spp., *Zannichellia* spp.), can grow only in aquatic or riverine environments such as watercourses, canals or ponds: these were primarily found in Ferrara (which is touched by a wide navigable canal with a dock) and Reggio Emilia, and secondarily in Bologna, Forlí and Ravenna (Table 3). Their absence in the city of Parma, which is crossed by the Parma and Baganza torrents, can be explained only by thinking of the still insufficient floristic exploration: this is, in fact, the city with the lowest species richness among those analysed (Table 1).

### 3.4. Halophilous Species

In urban contexts, the presence of halophilous species is mostly linked to the common practice of spreading salt on the roads during winter to delay rainfall water freezing [100]. Many of the halophilous species observed in this study are indeed myo-halophilous or facultative halophilous species (e.g., *Dittrichia graveolens* (L.) Greuter, *Erodium laciniatum* (Cav.) Willd., *Hordeum marinum* Huds., *Plantago coronopus* L.) which are actually typical of badlands and seaside areas with sandy substrates and brackish humid soils [77]: the use mentioned above allows a certain (weak) salt tenor to accumulate in the soil of flowerbeds, lawns, road margins, fissures of sidewalks, etc., so that these species can grow and propagate for years.

The true halophilous species, instead, are confined to the sole town of Ravenna, because it is the only one of the cities analysed which has a port upon the sea and some wetlands with marine water included within the urban area: in this case, species like *Carex extensa* Gooden., *Halimione portulacoides* (L.) Aellen. or *Salicornia patula* Duval-Jouve may be found in the port zone or in some relictual areas of coastal salt marshes [101].

### 3.5. Allochthonous Species

The average incidence of exotic species in the 7 cities is 25.6%, whereas in the entirety of Emilia-Romagna it is only 17.6% [61,62]. Only in Ferrara and Reggio Emilia is their presence (19.9 and 18.2%, respectively) substantially equivalent to the regional one, which means that most cities proportionally host more exotic species than a region 22510 km^2^ wide, and, therefore, they can effectively act as a spreading source for new, sometimes undesirable entities. In Bologna, the allochthonous presence even surpasses 30%, which can be explained by the city’s role as a railway and highway hub of international importance [102]. In this scenario, archaeophytes (the anthropophilous species of most ancient introduction, not by chance regarded by some authors as “honorary natives” [103]) are a very marginal presence, always limited to 4–5% of the total list, whereas neophytes (no matter if they behave as casual, naturalised or invasive) are the large majority, both in every city and in the global flora. Hence, it is not surprising that many of the newly discovered species for the regional or national flora are neophytes, principally cultivated as ornamental or food plants. In this sense, the role of infrastructure must not be underestimated: the railway yard of San Donato hosts 5 new species on its own [59], a situation partially confirmed (even if on a lesser scale) in the station of Parma (1 new species: see [57,104]). Again, in a European context (Serbia), but with notably smaller cities on average (a few tens of thousands of inhabitants), the allochthonous species presence is 15.2% of the total list, oscillating between 8 and 23% of the flora of the single towns [43]; even in the largest of them, the allochthonous species do not surpass 17% of the city’s flora. This can be explained in various ways: the lesser importance (from a traffic viewpoint) of the transport infrastructures connecting the cities analysed, the less rich flora of the territory considered [105], the different study methods adopted in the various cities [43], an insufficient floristic exploration, and so on. In other areas of continental Europe, instead, where the economic development and the movement of freight and people is very intense, the presence of allochthonous species is much higher: 40% of the list in Berlin [106] and in 54 other central European cities [28] and 45% (average value) in 19 towns in European Russia [33]. This fact is probably due to the absence of Mediterranean species in these environments, which occupy at least part of the ecological niches that thermophilous exotic species may colonise, as can be deduced by the comparison of the floras of some towns in central and southern Italy [20] and in Poland [107,108].

Concerning the geographical origin of the allochthonous species, as can be easily imagined, archaeophytes mostly come from SE-Europe, the Near East or central Asia; the Mediterranean basin also provides a significant part of these exotic species which arrived in Italy during Antiquity. Tropical or, however, thermophilous species are present, too (see Figure 6), native to Africa or the tropical regions of Asia. Neophytes, instead, mostly come from the New World (44.8%), as is logical when thinking of the progressive shifting of trade routes from the Levant (in the epoch of the Republic of Venice) to the Americas, from the late 16th century onwards. This phenomenon is particularly evident from the 1950s to the present day [109,110] due to the great agricultural, economic and trade changes that occurred after the Second World War (e.g., [83]).

Floristic pollution is particularly evident along transport infrastructures. The railway flora is a classic example in this sense, since it clearly shows an ongoing transformation: we remember, for example, the presence of *Cenchrus longispinus* (Hack.) Fernald in Bologna (where it arrived from the Adriatic coast), *Oxybaphus nyctagineus* in the freight yards of San Donato and Parma [57,58] and *Epilobium brachycarpum* (again in the freight yard of San Donato), which is present only in railway contexts at a European level [56]. Among the species native to Italy, but typical of environments differing from those of the reference territory (i.e., the so-called local allochthonous species), we may cite *Tribulus terrestris* L. in almost all of the stations of the Piacenza–Rimini railway (where it arrived moving from the Adriatic coast), or *Atriplex tatarica* L. along the Milan–Naples highway (from the Parma province to the Bologna apennines), the Parma–La Spezia highway (from Parma to Fornovo di Taro), the Bologna–Padova highway (from Bologna to Ferrara) and the ring roads of Modena and Bologna (where it can establish itself thanks to the use of spreading salt on the roads in the cold seasons [100]).

### 3.6. Species New for the National or Regional Flora

In a territory quite well-known from a floristic viewpoint such as Emilia-Romagna, given that a more or less systematic naturalistic exploration already began in the 16th century with Luca Ghini and Ulisse Aldrovandi [110,111,112,113,114,115,116,117] and has constantly continued in the following centuries (e.g., [118,119,120]), the discovery of new species typical of the middle latitudes and continental climates is not fully unlikely, but surely infrequent. Not by chance, most of the newly discovered species, both at the regional and national levels, are neophytes (24 out of 32; see Table S3), which arrived in Emilia-Romagna thanks to commerce, gardening, the movement of people from one country to another and not rarely from continents other than Europe; in many cases, these newcomers do not have particular aesthetic value (*Amaranthus* spp., *Eragrostis* spp., etc.), so their import is accidental and involuntary. In other cases, these novelties are ornamental or food species (in both cases mostly exotic) escaped from cultivation (e.g., *Cotoneaster* spp., *Gazania* gr. *hybrida*, *Solanum melongena*). Finally, there are also a few cases of species typical of Mediterranean Italy [77], of the warmest and driest parts with a subtropical climate (for example, *Ceratonia siliqua*), which could establish in some peculiar environments such as the railway yards, the urban city walls or the historical centres, thanks to the suitable microclimate of these places (e.g., [86,104,121,122,123]) and the constant arrival of new propagules, at least for a certain time. As can be seen, most (if not all) of the new species are allochthonous and thermophilous and native to the tropical or intertropical regions of the world (see Figure 6 and [124]): the role of global warming and the urban heat island must not be underestimated when reasoning about the possibility of the colonisation of an exotic species in an urban environment (e.g., [125]). It has been demonstrated that, with respect to the peri-urban country areas, in cities of average dimensions (about 150–200,000 inhabitants) the urban heat island raises average monthly temperatures by 1.4 °C during the day and by over 6 °C in the night hours, especially during peaks of heat and cold [123]; in cities of about 400,000 inhabitants, the urban heat island raises average minimum temperatures by 3.5 °C, where average temperature differences between the urban area and the surrounding countryside are 1.5–2 °C during the daytime and *circa* 6.5 °C during the night [126]. All of this greatly reduces the days with maximum temperatures equal to zero or less (on average 1.2 per year in Modena, considering the climatic reference period 1991–2020 [127]), therefore allowing the survival and spreading of species typical of warmer climates.
***An “archaeobotanical window” for three cities*** For over 20 years, the Laboratory of Palynology and Palaeobotany of the University of Modena and Reggio Emilia has performed archaeobotanical analyses (seeds/fruits, woods/charcoals, pollen/spores, NPPs) in Emilia-Romagna urban sites (Modena, Ferrara, Parma, Faenza, Forlí, Reggio Emilia, Ravenna, Argenta, Lugo and Imola—with published and unpublished data). In many of the investigated cities, in the initial phases of their foundation many signs of shallow water and humid environments were detected, which can last for a longer or shorter period, sometimes diminishing and modifying while the urban centre enlarges and becomes more complex. Archaeobotanical records show an excellent biodiversity of wetland plants. In the case of Modena, this biodiversity decreased during the flood phase (especially between the 5th and 7th century CE), despite wetlands still expanding notably and taking over, as is easily understandable. Therefore, because of these important events, at least in the first period, the territory seemed to undergo a sort of homogenization with respect to former centuries when it appeared extremely differentiated within the urban and peri-urban space [92]. For Parma, we have archaeobotanical analyses of the site of Piazza Garibaldi, today within the historical city centre. In the phase preceding the town’s foundation (4th–3rd century BCE), it is believed that the area was used for ritual purposes. This hypothesis is confirmed by archaeobotanical evidence, too, and signs of various spontaneous synanthropic species are present. Weeds show a differentiation of environments already characterised by human presence, such as trampled zones, uncultivated lands, margins, cultivated fields and zones with an accumulation of nitrates [128]. During the Mediaeval period (10th–11th century CE), the investigated space was occupied by the city market. Synanthropic species increase in quantity and quality, and weeds and commensal species of fields and kitchen gardens become more evident (with species that are rare today, such as *Agrostemma githago* L. and *Thymelaea passerina* (L.) Coss. et Germ.), like nitrophilous species [128]. For Modena, a Roman colony founded, with Parma, in 183 BCE, 12 urban and peri-urban sites were studied (excluding the funerary only contexts); these sites cover a wide chronological range, spanning from the 2nd century BCE to 13th century CE. The Roman/Late Antiquity period was treated and studied in depth (see [129,130,131]), whereas for the Mediaeval one (e.g., [132]) there are many data still partially or totally unedited. A first attempt of comparison on archaeobotanical evidence of weeds between the Roman and Mediaeval periods was performed on four sites within the current historical city centre. According to this preliminary analysis, during the Roman period the species list of this category was practically double that in the Middle Ages. Species like *Salvia pratensis* L., *Sanguisorba minor* Scop. and *Reseda luteola* L., which were linked to sheep farming and its related industries, nearly disappear in the Middle Ages, when hygrophilous species such as *Potentilla reptans* L. or *Persicaria lapathifolia* (L.) Delarbre became widespread and numerous, expanding after the important flood events that hit the city. In the Mediaeval period, clearly nitrophilous species seem to increase, probably because of more intense urbanisation and difficulties in managing waste disposal. As seen in Parma, commensal (or invasive) species of cultivated lands, such as *Conringia orientalis* (L.) Dumort., *Neslia paniculata* (L.) Desv., etc., become evident in the past of Modena, whereas today they have practically disappeared even in the rural zones of the surrounding territory [133]. Ferrara, a town of early-Mediaeval foundation, gave a large amount of archaeobotanical remains, especially in five urban contexts of today’s historical city centre, which can be dated between the mid-10th and early-16th century CE. The strong link between Ferrara and the Po river is visible in the archaeological deposits, where many species typical of wetlands and shallow waters are testified. On a regional scale, many of these species have now disappeared, or are at least protected (e.g., *Schoenoplectus supinus* (L.) Palla, *Nuphar lutea* (L.) Sm., *Nymphaea alba* L., *Nymphoides peltata* (S.G. Gmel.) Kuntze), whereas during the Renaissance some were used even as ornamental plants in elegant settings in the heart of the city [134]. Here, too, as in other Emilia-Romagna cities investigated for their Middle Ages archaeobotanical remains, the taxa indicators of nitrified soils and commensal of cultivated fields are numerous (e.g., *Chenopodium* spp., *Hyoscyamus niger* L., *Conium maculatum* L., *Myagrum perfoliatum* L.). The ancient maps depict Ferrara as a true “patchwork” of empty spaces (squares and courtyards, but also gardens, kitchen gardens and orchards) and full areas (buildings), a situation that allowed a notable weed biodiversity to develop, exactly the kind of diversified environments available to urban flora [135,136,137,138,139].

## 4. Materials and Methods

### 4.1. Study Area

This study takes into account 7 towns in northern Italy, all of them belonging to the Emilia-Romagna region in the southern part of the Po Plain: from west to east, Parma, Reggio Emilia, Modena, Bologna, Ferrara, Forlí and Ravenna (Figure 1; Table 11). Inhabitants vary from around 117,000 in Forlí up to ca. 390,000 in Bologna; altitude spans from 4 to 57 m a.s.l. (Ravenna and Parma, respectively). Parma is crossed by the Parma and Baganza torrents, Reggio Emilia is crossed by the Cròstolo stream, Modena is flanked by the Secchia and Panaro rivers, Bologna is crossed by some canals (formerly streams) and is touched by the Reno river and the Sàvena torrent, Ferrara is touched by a navigable canal (Po di Volano, formerly a branch of the Po delta), Forlí is crossed by the Montone river and flanked by the Ronco river, and Ravenna has a port on the Adriatic Sea and some wide brackish coastal marshes (the so-called *pialasse*) within the periphery urban tissue.

According to Köppen [81], the climate of these towns is temperate-humid (*Cfa*).

Modena and Bologna (and probably Ravenna, too) were founded by the Etruscans, and Forlí, Parma and Reggio Emilia by the Romans, whereas Ferrara is of Mediaeval origin.

The historical, cultural and political importance of many of these towns is relevant, even at a continental scale. Ravenna was the capital of the Western Roman Empire (years 402–476 CE), the Ostrogothic Kingdom (years 493–540 CE) and the Exarchate of Italy (years 584–751 CE). Bologna was, for more than a millennium, one of the most important towns of the State of the Church (it was second to Rome, only) and surely the most vivacious cultural and industrial centre; since the Middle Ages it has been a renowned university town, hosting the most ancient European university [140]. Ferrara was the capital of the Este Duchy (years 1471–1598) and, during the Renaissance, hosted one of the most rich and enlightened courts of Europe, where fine arts, sciences and commerce were greatly supported and encouraged, and patronage was common [141,142]. Modena and Parma, in turn, were the capitals of the Duchy of Modena and Reggio (years 1598–1859) and the Duchy of Parma and Piacenza (years 1545–1859), both characterised by an Austro-Hungarian influence during the 18th and especially 19th centuries, with consequent scientific and cultural development [143,144]. For this illustrious past, Ravenna, Modena and Parma are adorned with notable monuments and architectures, which are in some cases listed among the UNESCO World Heritage [145,146]; the historical centres of Bologna and Ferrara are, in turn, ascribed to the UNESCO World Heritage for their artistic and architectonic value [147,148].

For all of these reasons, we can assume that the 7 cities here treated are representative of the climatic and environmental conditions which can be found in the principal towns of Emilia-Romagna.

**Table 11:** A synopsis of the principal characteristics of the towns considered in this study.

Town	Geographical Coordinates	Inhabitants (31 August 2024) *	Altitude (m a.s.l.)	Parts of the Urban Area Considered Here
Bologna	Lat. 44° 29′ N Long. 11° 20′ E	390,850	54	Historical city centre, peripheries, industrial areas, railways and railway areas, cemetery, canals
Ferrara	Lat. 44° 50′ N Long. 11° 37′ E	129,427	9	Historical city centre, peripheries, industrial areas, railways and railway areas, cemetery, canals
Forlí	Lat. 44° 13′ N Long. 12°0 2′ E	117,464	34	Historical city centre, peripheries, industrial areas, railways and railway areas, cemetery, Ronco and Montone rivers
Modena	Lat. 44° 38′ N Long. 10° 55′ E	185,180	34	Historical city centre, peripheries, industrial areas, railways and railway areas, cemetery
Parma	Lat. 44° 48′ N Long. 10° 19′ E	198,953	57	Historical city centre, peripheries, industrial areas, railways and railway areas, cemetery
Ravenna	Lat. 44° 24′ N Long. 12° 12′ E	156,551	4	Historical city centre, peripheries, industrial areas, railways and railway areas, cemetery, port, canals, urban wetlands
Reggio Emilia	Lat. 44° 42′ N Long. 10° 38′ E	172,182	56	Historical city centre, peripheries, industrial areas, railways and railway areas, cemetery, T. Cròstolo

* Data retrieved from ISTAT [149]. The number refers to the entire municipality territory, which is a good representation of the total number of people which are present daily in the urban area, including the flow of commuters (that is estimable in the tens of thousands of people every day in every city, coming from the province or the neighbouring provinces).

### 4.2. Data Extraction and Preparation

#### 4.2.1. Data Collection

For every town considered in this study, floristic data were taken from the Floristic Database of the Emilia-Romagna Region, managed by one of the authors (A.A.). Both published and unpublished data were used (see Table 12 for more details), referring to the current period (years 1990–2024). Indeed, more or less satisfying investigations on urban flora were already performed in Bologna [59,86,98,150,151], Ferrara [152,153], Modena [54,87,133], Forlí [154] and Ravenna [101], whereas for others (e.g., Piacenza, Cesena and Rimini) data collection was still insufficient to justify inclusion in such a synthesis. For every town, a specific floristic list was prepared, summing up all of the available information, both published and unpublished: the completeness of the floristic lists, therefore, depended first on the intensity and time duration of the floristic exploration itself.

Only the areas which could be regarded as truly urban were taken into account: historical city centres, cemeteries, residential peripheries of various ages, industrial areas (both in use and abandoned), parking areas, railways (only the segments included in the urban area) and railway areas (stations, depots, freight yards), ports, urban parks and gardens (the so-called urban green), botanical gardens, flowerbeds, flowerpots used as street furniture and rivers and canals (only the segments included in the urban area). In these areas, only spontaneous species were recorded, systematically excluding all of the individuals cultivated for various reasons; cultivated species were considered only in cases of spontaneous dissemination [99,155]. Worksites and private yards and gardens were not included. Despite the notable botanical interest (e.g., [156]), for the moment, airports were not analysed because of the particular security constraints to be faced in view of such an investigation.

The agricultural areas included in the urban perimeter were not taken into account to avoid the insertion of floristic elements extraneous to the true urban environments [99].

**Table 12:** A synopsis of the data sources (both published and unpublished) used in this study. The number of contributors (i.e., persons involved in the floristic exploration of the towns considered) is also reported.

City	Source	Type of Urban Environment	Number of Contributors
Bologna	Salinitro et al. [86,150]	Historical centre	3
	Salinitro et al. [98]	Green areas (hydro-hygrophilous species)	
	Alessandrini [59]	Railway freight yard	
	Alessandrini and Trentanovi [151]	Prati di Caprara	
	Alessandrini, unpubl.	Entire town	
Ferrara	Alessandrini and Pellizzari, unpubl.	Jewish cemetery	3
	Alessandrini and Pellizzari, unpubl.	Monumental cemetery (Certosa di Ferrara)	
	Pellizzari, *in litteris*	Entire town	
	Piccoli [152]	City walls	
	Pellizzari et al. [153]	Entire town	
Forlí	Bugni et al. [154]	Entire town	8
	Frascari, unpubl.	Urban cemeteries	
	Sirotti, unpubl.	Entire town	
Modena	Buldrini et al. [54]	Historical centre	3
	Buldrini et al. [87]	Monumental cemetery	
	Costa [157]	19th cent. residential and industrial periphery	
	Gruppi, unpubl.	Ex railway path	
	Alessandrini et al., unpubl.	Entire town	
Parma	Adorni and Ghillani, unpubl.	Entire town	2
	Adorni and Ghillani [57,104]	Railway station	
Ravenna	Lazzari et al. [101]	Entire town	6
	Alessandrini, Montanari, Faggi et al., unpubl.	Entire town	
Reggio Emilia	Morelli, unpubl.	Entire town	3

#### 4.2.2. Data Treatment

For every species, life forms and chorotypes were attributed according to Pignatti et al. [77], with integrations by POWO [124]; in view of the analyses, chorotypes were subsequently grouped into macro-chorotypes, as already undertaken in former works [83,109,158,159,160]. For the autochthonous species, the following macro-chorotypes were adopted:

- Boreal: species native to the cold regions of Eurasia and northern America
- Cosmopolitan: cosmopolitan or sub-cosmopolitan species
- Eurasian: species of the temperate and sub-steppic regions of Eurasia
- Exotic: all species which are not native to the Italian territory
- Mediterranean: species native to the Mediterranean basin
- Southern European orophytes: species of the mountain ranges of southern Europe
- Cultivated: species existing only in cultivation (stabilised hybrids, sterile cultivars, etc.)

For the allochthonous species, instead, the following macro-chorotypes were adopted:

- Boreal: species of the cold regions of Eurasia and northern America
- Cosmopolitan: cosmopolitan or sub-cosmopolitan species
- Eurasian: species of the temperate and sub-steppic regions of Eurasia
- Tropical Asian: species of the tropical regions of Asia and Indian subcontinent
- Mediterranean: species native to the Mediterranean basin
- Southern European orophytes: species of the mountain ranges of southern Europe
- Northern African: species of the temperate regions of northern Africa
- Tropical African: species of the tropical and intertropical parts of Africa
- Saharo-Sindian: species of the deserts from northern Africa and India
- Southern African: species of the temperate regions of southern Africa (here including the Cape province, with a Mediterranean-type climate)
- Northern American: species of the temperate regions of northern America
- Neotropical: species of the tropical and intertropical regions of the Americas
- Southern American: species of the temperate regions of southern America
- Panamerican: species of the temperate regions of the Americas
- Pantropical: species of the tropical and intertropical regions of the world
- Australian: species native to the Australian continent
- Malagasy: species native to Madagascar
- Cultivated: species existing only in cultivation (stabilised hybrids, sterile cultivars, etc.)

Allochthonous species, considered as such on account of their geographical origin, were divided into archaeophytes and neophytes following Galasso et al. [62], but treated separately to better understand the presence and impact on the flora of every town of the most ancient anthropophilous species here recorded (the archaeophytes) and of the species of recent ingression due to the development of new trade routes, commercial exchanges and human migrations (the neophytes). To avoid confusion in the evaluation of the floristic pollution, ergasiophytes (*sensu* [27]) were not separated from autochthonous species.

Every species was attributed to a phytosociological class according, wherever possible, to various bibliographical sources [63,77,161,162,163,164,165,166,167,168], or was added by one of the authors (M.A.) based on his own knowledge of the regional territory; when a species could not be clearly ascribed to a precise class, it was regarded as a “casual sporadic” (in many cases, they were allochthonous cultivated species which rarely naturalise—often near the mother plants—and can behave as adventitious in vegetation contexts of not univocal interpretation). Then, the phytosociological spectrum was calculated, grouping the classes based on their ecological characteristics (e.g., halophilous vegetation, woody riparian formations, humid grasslands, etc.); this grouping was performed according to [63].

Ellenberg’s bioindication values corrected for the Italian flora were also added to every species, according to [169,170,171].

Nomenclature follows Pignatti et al. [77].

### 4.3. Data Analysis

The following analyses were performed:

- Rarity of the species and their affinity to urban environments (“urbanophily”), based on their presence in the towns considered: presence in 7 towns—steno-urbanophilous species; presence in 5 or 6 towns—urbanophilous species; presence in 4 towns—urban-neutral species; presence in 2 or 3 towns—urbanophobous species; presence in 1 town—steno-urbanophobous species.
- Biological spectrum.
- Chorological spectrum, paying special attention to the exotic species.
- Phytosociological spectrum, calculated on the classes to which every species was referred. For the environmental macro-types most represented in the urban flora (i.e., the physiognomical macro-categories of vegetation), the frequency of the classes of synanthropic vegetation was calculated first for the global flora and then for constant species only; the results were finally compared.
- Presence of hydro-hygrophilous species in urban environments and their relationship with the eventual presence of watercourses crossing the town. As performed in previous works [109,172], these species were defined as those with an Ellenberg’s bioindication value for soil moisture U ≥ 7: U = 7 and 8, hygrophilous species; U = 9 and 10, palustrine species; U = 11 and 12, aquatic species (hydrophytes).
- Presence of halophilous or however salt-tolerant species, defined as those with an Ellenberg’s bioindication value for soil salinity S ≥ 1: S = 1, myo-halophytes; S = 2, facultative halophytes; S = 3, true halophytes.
- Insights into the steno-urbanophilous species.

## 5. Conclusions

As previously illustrated, a notable biological richness is contained in the cities analysed, despite the constant and heavy (although irregular in time and space) human pressure and impact: this fact is related both to the biological diversity typical of Italy and to the considerable economic development of the area investigated. The allochthonous species are one of the main components of urban flora, but with a lesser percentage than in other climatic and environmental contexts, such as central Europe; thus, the cities analysed may be placed in an intermediate situation between central European and Mediterranean ones, which show even lower values of floristic pollution. Despite this, of the species that are new for the Italian or regional flora discovered during this study, the largest part is made up of exotic entities. The percentage of protected species is minimal, but their presence is a clear sign of the importance of artificial environments (especially marginal and abandoned intra-urban or peri-urban areas) for the conservation of biological diversity. The urban ecosystem has therefore proved again to be a mosaic of very different habitats, characterised by a rich, diversified and mutable spontaneous flora.

This study is the result of more than 30 years of floristic exploration of the territory, performed by both academic and amateur botanists; the contribution of the latter was fundamental and irreplaceable in collecting the very large amount of data analysed. Despite this, the current scientific tendency is to abandon field research, regarded as expensive, time-consuming and not able to assure an adequate editorial collocation of the articles produced [173,174]. Field studies and floristic exploration are instead an essential base to correctly understand the evolution of biological diversity in time and space, especially in today’s perspective of global change. We hope that our contribution can help recognise the importance of this part of the botanist’s work and may stimulate other people to continue the centuries-old tradition of nature exploration in Emilia-Romagna, for a better understanding of plant species distribution dynamics in a more and more globalised world.

## Figures and Tables

**Table 1:** Number of species recorded in every city.

City	Number of Species Recorded	% of Species Out of the Total
Bologna	754	57.7
Ferrara	523	40.0
Forlí	756	57.8
Modena	606	46.4
Parma	432	33.1
Ravenna	603	46.1
Reggio Emilia	656	50.2

**Table 2:** The scheme of the macro-categories of vegetation detectable based on the total floristic list (1305 species). The number of classes attributable to every macro-category is also reported.

Physiognomic Macro-Category	Synthetic Description	Number of Classes in the Italian Vegetation	Number of Classes Attributed to the Urban Flora
Mobile and fixed aquatic vegetation	aquatic	11	2
Amphibian vegetation of rivers, springs and marshes	amphibian	8	5
Halophilous vegetation of coasts, inland areas and sand dunes	halophilous/ psammophilous	9	5
Chasmophytic, glareicolous and epiphytic vegetation	chasmophytic/ glareicolous	5	5
Herbaceous synanthropic, ruderal and tall forbs vegetation	synanthropic	11	7
Suprasylvatic cryophilous vegetation and of cryo-turbed soils	suprasylvatic	5	0
Grassland vegetation	grassland	9	7
Lands, garrigues and shrub belt vegetation	shrub	6	5
Forest and pre-forest vegetation	forest	11	7

**Table 3:** The frequency of the physiognomic macro-categories of vegetation in the cities analysed and in the total flora. The values are expressed as a percentage.

Macro-Category	Total Flora	Bologna	Ferrara	Forlí	Modena	Parma	Ravenna	Reggio Emilia
aquatic	1.7	0.6	2.6	0.6	0.2	0.0	0.8	1.3
amphibian	7.1	3.9	5.7	6.0	2.6	2.4	6.5	7.5
halophilous/ psammophilous	1.7	0.1	0.2	0.4	0.2	0.0	3.1	0.2
chasmophytic/ glareicolous	3.7	4.9	4.1	2.5	3.8	2.1	3.1	3.1
synanthropic	44.6	50.4	49.5	50.2	53.3	54.5	49.1	47.5
grassland	23.6	20.5	23.6	22.4	24.0	20.9	22.6	24.1
shrub	4.6	5.8	3.9	5.1	4.9	5.9	4.6	4.4
forest	13.1	13.7	10.4	12.8	11.1	14.2	10.4	11.9

**Table 4:** The frequency of the classes of synanthropic vegetation calculated on the total floristic list (1305 species) and for the constant species only. The values are expressed as a percentage.

Phytosociological Class	Total Flora	Constant Species
*Artemisietea vulgaris* Lohmeyer, Preising & Tüxen ex Von Rochow 1951	21.0	24.1
*Filipendulo ulmariae-Convolvuletea sepium* Géhu & Géhu-Franck 1987	5.9	3.8
*Galio aparines-Urticetea dioicae* Passarge ex Kopecký 1969	6.0	9.0
*Pegano harmalae-Salsoletea vermiculatae* Br.-Bl. & O. Bolòs 1958	0.2	0.0
*Polygono arenastri-Poetea annuae* Rivas-Martínez 1975 *corr.* Rivas-Martínez, Báscones, T.E. Diáz, Fernández-González & Loidi 1991	4.4	5.3
*Stellarietea mediae* Tüxen, Lohmeyer & Preising ex Von Rochow 1951	57.8	55.6
*Trifolio medii-Geranietea sanguinei* Müller 1962	4.8	2.3

**Table 5:** The frequency of the classes of synanthropic vegetation calculated for the total floristic list (1305 species) and for every city analysed. The values are expressed as a percentage. Abbreviations: AV—*Artemisietea vulgaris*; FC—*Filipendulo ulmariae-Convolvuletea sepium*; GU—*Galio aparines-Urticetea dioicae*; PS—*Pegano harmalae-Salsoletea vermiculatae*; PP—*Polygono arenastri-Poetea annuae*; SM—*Stellarietea mediae*; TG—*Trifolio medii-Geranietea sanguinei*.

Phytosociological Class	Total Flora	Bologna	Ferrara	Forlí	Modena	Parma	Ravenna	Reggio Emilia
AV	21.0	22.1	22.6	21.5	20.5	23.5	21.5	23.4
FC	5.9	4.3	6.7	5.6	4.9	4.3	6.6	7.6
GU	6.0	7.5	7.1	6.8	6.5	9.1	6.9	7.9
PS	0.2	0.3	0.0	0.3	0.0	0.0	0.0	0.0
PP	4.4	5.5	5.2	4.3	5.9	5.7	4.8	4.0
SM	57.8	57.2	53.6	58.0	59.3	53.0	57.1	52.8
TG	4.8	3.2	4.8	3.5	2.9	4.3	3.1	4.3

**Table 6:** The frequency of the classes of grassland vegetation calculated on the total floristic list (1305 species) and for the constant species only. The values are expressed as a percentage.

Phytosociological Class	Total Flora	Constant Species
*Agrostietea stoloniferae* Oberdorfer 1983	9.3	19.5
*Festuco valesiacae-Brometea erecti* Br.-Bl. & Tüxen ex Br.-Bl. 1949	23.9	14.6
*Koelerio glaucae-Corynephoretea canescentis* Klika in Klika & V. Novák 1941	12.5	17.1
*Lygeo sparti-Stipetea tenacissimae* Rivas-Martínez 1978	1.7	0.0
*Molinio-Arrhenatheretea* Tüxen 1937	34.3	41.5
*Sedo albi-Scleranthetea biennis* Br.-Bl. 1955	2.4	2.4
*Tuberarietea guttatae* (Br.-Bl. in Br.-Bl., Roussine & Nègre 1952) Rivas Goday & Rivas-Martínez 1963	15.9	4.9

**Table 7:** The frequency of the classes of grassland vegetation calculated for the total floristic list (1305 species) and for every city analysed. The values are expressed as a percentage. Abbreviations: AS—*Agrostietea stoloniferae*; FB—*Festuco valesiacae-Brometea erecti*; KC—*Koelerio glaucae-Corynephoretea canescentis*; LS—*Lygeo sparti-Stipetea tenacissimae*; MA—*Molinio-Arrhenatheretea*; SS—*Sedo albi-Scleranthetea biennis*; TU—*Tuberarietea guttatae*.

Phytosociological Class	Total Flora	Bologna	Ferrara	Forlí	Modena	Parma	Ravenna	Reggio Emilia
AS	9.3	11.3	15.8	13.1	13.8	14.8	13.5	14.3
FB	23.9	15.5	20.8	23.3	18.8	18.2	15.8	26.6
KC	12.5	19.0	15.0	13.1	18.1	17.0	15.0	11.7
LS	1.7	2.1	0.0	1.7	2.2	1.1	2.3	2.6
MA	34.3	35.2	39.2	35.2	35.5	40.9	39.8	36.4
SS	2.4	3.5	3.3	1.1	2.9	2.3	2.3	1.9
TU	15.9	13.4	5.8	12.5	8.7	5.7	11.3	6.5

**Table 8:** The number of hydro-hygrophilous species recorded in every city. The percentage is calculated from the total flora of every city (see Table 1), summing the three categories (hygrophilous, palustrine, hydrophilous).

City	Hygrophilous Species	Palustrine Species	Aquatic Species	% of Species
Bologna	67	17	6	11.9
Ferrara	53	24	14	17.4
Forlí	71	27	5	13.6
Modena	58	16	2	12.5
Parma	39	4	0	10.0
Ravenna	73	27	7	17.7
Reggio Emilia	70	32	13	17.5
*Total species number*	*130*	*57*	*26*	*16.3*

**Table 9:** The number of halophilous species recorded in every city. The percentage is calculated from the total flora of every city (see Table 1), summing the three categories (myo-halophytes, facultative halophytes, true halophytes).

City	Myo-Halophytes	Facultative Halophytes	True Halophytes	% of Species
Bologna	14	2	0	2.1
Ferrara	7	1	0	1.5
Forlí	14	5	0	2.5
Modena	11	2	0	2.1
Parma	2	0	0	0.5
Ravenna	17	4	15	6.0
Reggio Emilia	10	3	0	2.0
*Total species number*	*28*	*7*	*15*	*3.8*

**Table 10:** The presence of allochthonous species in the analysed cities. The percentages of archaeophytes and neophytes are calculated from the total number of species; within brackets, the percentage calculated from the percentage of allochthonous species for each town is also reported.

City	Total Number of Species	% of Allochthonous Species	% of Archaeophytes	% of Neophytes
Bologna	754	30.9	5.2 (16.7)	25.8 (83.3)
Ferrara	523	19.9	3.8 (19.2)	16.1 (80.8)
Forlí	756	23.2	5.3 (22.7)	17.9 (77.3)
Modena	606	23.4	5.6 (23.9)	17.8 (76.1)
Parma	432	22.7	5.3 (23.5)	17.4 (76.5)
Ravenna	603	19.5	4.5 (23.1)	15.0 (76.9)
Reggio Emilia	656	18.2	4.1 (22.7)	14.1 (77.3)
*Total floristic list*	*1305*	*26.8*	*5.2*	*21.6*

## Data Availability

Data supporting reported results are provided in the electronic Supplementary Materials.

## Supplementary Materials

The following supporting information can be downloaded at: https://www.mdpi.com/article/10.3390/plants14030450/s1, Table S1: A list of the taxa found, with family, presence in cities (indicated with 1), number of cities of presence, regional (Emilia-Romagna region) protection, notes on nomenclature and taxonomy; Table S2: List of allochthonous species: archaeophytes and neophytes; Table S3: A list of the species new for the flora of the Emilia-Romagna region which were found during the research on urban flora, with an indication of city and status (native or alien).
